# Persistent erectile dysfunction in men exposed to the 5α-reductase inhibitors, finasteride, or dutasteride

**DOI:** 10.7717/peerj.3020

**Published:** 2017-03-09

**Authors:** Tina Kiguradze, William H. Temps, Paul R. Yarnold, John Cashy, Robert E. Brannigan, Beatrice Nardone, Giuseppe Micali, Dennis Paul West, Steven M. Belknap

**Affiliations:** 1 Department of Dermatology, Feinberg School of Medicine, Northwestern University, Chicago, IL, USA; 2 Optimal Data Analysis LLC, San Diego, CA, USA; 3 Department of Urology, Feinberg School of Medicine, Northwestern University, Chicago, IL, USA; 4 Department of Medicine, Division of General Internal Medicine and Geriatrics, Feinberg School of Medicine, Northwestern University, Chicago, IL, USA; 5 Department of Dermatology, Faculty of Medicine and Surgery, University of Catania, Catania, Italy

**Keywords:** Finasteide, Dutasteride, Persistent sexual dysfunction, Impotence, Low libido, Pharmacoepidemiology, Drug safety

## Abstract

**Importance:**

Case reports describe persistent erectile dysfunction (PED) associated with exposure to 5α-reductase inhibitors (5α-RIs). Clinical trial reports and the manufacturers’ full prescribing information (FPI) for finasteride and dutasteride state that risk of sexual adverse effects is not increased by longer duration of 5α-RI exposure and that sexual adverse effects of 5α-RIs resolve in men who discontinue exposure.

**Objective:**

Our chief objective was to assess whether longer duration of 5α-RI exposure increases risk of PED, independent of age and other known risk factors. Men with shorter 5α-RI exposure served as a comparison control group for those with longer exposure.

**Design:**

We used a single-group study design and classification tree analysis (CTA) to model PED (lasting ≥90 days after stopping 5α-RI). Covariates included subject attributes, diseases, and drug exposures associated with sexual dysfunction.

**Setting:**

Our data source was the electronic medical record data repository for Northwestern Medicine.

**Subjects:**

The analysis cohorts comprised all men exposed to finasteride or dutasteride or combination products containing one of these drugs, and the subgroup of men 16–42 years old and exposed to finasteride ≤1.25 mg/day.

**Main outcome and measures:**

Our main outcome measure was diagnosis of PED beginning after first 5α-RI exposure, continuing for at least 90 days after stopping 5α-RI, and with contemporaneous treatment with a phosphodiesterase-5 inhibitor (PDE_5_I). Other outcome measures were erectile dysfunction (ED) and low libido. PED was determined by manual review of medical narratives for all subjects with ED. Risk of an adverse effect was expressed as number needed to harm (NNH).

**Results:**

Among men with 5α-RI exposure, 167 of 11,909 (1.4%) developed PED (persistence median 1,348 days after stopping 5α-RI, interquartile range (IQR) 631.5–2320.5 days); the multivariable model predicting PED had four variables: prostate disease, duration of 5α-RI exposure, age, and nonsteroidal anti-inflammatory drug (NSAID) use. Of 530 men with new ED, 167 (31.5%) had new PED. Men without prostate disease who combined NSAID use with >208.5 days of 5α-RI exposure had 4.8-fold higher risk of PED than men with shorter exposure (NNH 59.8, all *p* < 0.002). Among men 16–42 years old and exposed to finasteride ≤1.25 mg/day, 34 of 4,284 (0.8%) developed PED (persistence median 1,534 days, IQR 651–2,351 days); the multivariable model predicting PED had one variable: duration of 5α-RI exposure. Of 103 young men with new ED, 34 (33%) had new PED. Young men with >205 days of finasteride exposure had 4.9-fold higher risk of PED (NNH 108.2, *p* < 0.004) than men with shorter exposure.

**Conclusion and relevance:**

Risk of PED was higher in men with longer exposure to 5α-RIs. Among young men, longer exposure to finasteride posed a greater risk of PED than all other assessed risk factors.

## Introduction

There is conflicting information about sexual dysfunction associated with finasteride and dutasteride, the two 5α-reductase inhibitors (5α-RIs) marketed in the US. Isozymes 5α-R_1_ and 5α-R_2_ are expressed in adult male human prostate, epididymis, seminal vesicle, testis, genital skin, and brain (Aumüller et al., 1996; Hellwinkel et al., 2000; Nonomura et al., 1990; Thiele et al., 2005; Thigpen et al., 1993) and 5α-R_3_ is expressed ubiquitously in adult tissues (Godoy et al., 2011). Androgen receptors are expressed in both stromal and endothelial cells of adult male human corpus cavernosum (Schultheiss et al., 2003), which is functionally androgen-dependent in adult male humans (Rossi et al., 1998). Local conversion by 5α-R_1_ and 5α-R_2_ of testosterone to the more potent androgen, 5α-dihydrotestosterone (5α-DHT), is essential for development and maintenance of the normal structure and function of male reproductive tissues (Mahony et al., 1998; Oztekin et al., 2012; Tomada et al., 2013); local conversion by 5α-R_3_ of testosterone to 5α-DHT in adipose tissue (Fouad Mansour, Pelletier & Tchernof, 2016) contributes to systemic 5α-DHT levels.

Clinical development of finasteride followed observations that prepubertal castration prevents androgenic alopecia (Hamilton, 1942) and that male pseudohermaphrodites have nonpalpable prostates, scanty beards, and have neither androgenic alopecia nor acne (Imperato-McGinley et al., 1974; Imperato-McGinley & Peterson, 1976; Walsh et al., 1974). Pseudohermaphroditism is caused by loss-of-function mutation of the 5α-R_2_ gene, resulting in impaired conversion of testosterone to 5α-DHT, with varying degrees of genital ambiguity at birth. Pseudohermaphrodites are often raised as female but unexpectedly virilize at puberty, with partial masculinization of external genitalia (Imperato-McGinley et al., 1974; Sinnecker et al., 1996). The chemical composition-of-matter patent for finasteride describes finasteride and related compounds as “antiandrogenic by virtue of their ability to inhibit testosterone-5α-reductase” (Rasmusson & Reynolds, 1988). Exposure of men to finasteride mimics hereditary 5α-reductase deficiency by preferentially inhibiting 5α-R_2_ (Gormley et al., 1990), inducing a sex steroid profile “strikingly similar to that of pseudohermaphrodites” (Imperato-McGinley et al., 1990), with a 70% reduction in serum 5α-DHT (Drake et al., 1999; Grino, Griffin & Wilson, 1990; Marchetti & Barth, 2013; Roberts et al., 1999), corresponding to “circulating levels of 5α-DHT similar to those following castration” (Stoner, 1990). Dutasteride inhibits both 5α-R_1_ and 5α-R_2_, reducing serum 5α-DHT by 90% (Rittmaster et al., 2008).

There is conflicting information about the effect of 5α-RIs on testosterone in humans. Men exposed to finasteride had an increase over baseline of plasma testosterone levels; among men with baseline testosterone in the lowest tertile, plasma testosterone peaked at 1 year, then steadily declined, but did remain above baseline during the entire 4-year study (Roehrborn et al., 2003). Men with prostatic hyperplasia who were exposed to dutasteride had an increase over baseline of serum testosterone levels at one year (Hong et al., 2010). However, a long-term study found that men with prostatic hyperplasia who were exposed to finasteride had a progressive, and clinically significant, decline in testosterone over 45 months (Traish et al., 2015a). In men exposed to finasteride for one week, effective androgen levels in the prostate are similar to those in castrated men (Geller & Sionit, 1992). There is little available information about the effect of 5α-RI exposure on non-prostate genital tissues in humans. Chronic 5α-RI exposure in rats increases apoptosis and autophagy in corpus cavernosum smooth muscle, attenuating erectile function (Zhang et al., 2013). Given the pattern of expression and activity of androgen receptors and of 5α-reductases in multiple tissues and the marked effect of 5α-RIs on local and systemic 5α-DHT levels, it seems implausible that the anti-androgenic effects of 5α-RIs in men would be limited to prostate and scalp tissue.

The 5α-RIs also induce a global defect in C19 and C21 5α-metabolism, inhibiting 5α-reduction of progesterone, androstenedione, epitestosterone, cortisol, aldosterone, corticosterone, and deoxycorticosterone (Imperato-McGinley et al., 1985; Traish et al., 2015b) and thereby reducing levels of the brain neurosteroids that increase libido and sexual arousal (Finn et al., 2006; Melcangi et al., 2013; Torres & Ortega, 2003; Trost, 2013).

The FDA granted marketing approval for finasteride 5 mg in 1992, finasteride 1 mg in 1997, and dutasteride 0.5 mg in 2001. Per the full prescribing information (FPI), finasteride 5 mg is indicated for the treatment of symptomatic benign prostatic hyperplasia (BPH) in men with an enlarged prostate to improve symptoms, reduce the risk of acute urinary retention, and reduce the risk of the need for surgery including transurethral resection of the prostate (TURP) and prostatectomy. Per the FPI, finasteride 1 mg is indicated for the treatment of male pattern hair loss (androgenic alopecia) (Merck, 2014b). Per the FPI, dutasteride 0.5 mg is indicated for the treatment of symptomatic BPH in men with an enlarged prostate to improve symptoms, reduce the risk of acute urinary retention, and reduce the risk for the need for BPH-related surgery (GlaxoSmithKline, 2014a).

The FPI for finasteride 1 mg states that, regarding the clinical trial experience, “(t)here is no evidence of increased sexual adverse experiences with increased duration of treatment with (finasteride 5 mg)” and “resolution (of sexual adverse experiences) occurred in men who discontinued therapy with (finasteride 1 mg) due to these (sexual) side effects and in most of those who continued therapy” (Merck, 2014b). The FPI for finasteride 5 mg states that, regarding the clinical trial experience, “There is no evidence of increased sexual adverse experiences with increased duration of treatment with (finasteride 5 mg) (Merck, 2014c).” The FPI for dutasteride states, “In the three pivotal placebo-controlled BPH trials with (dutasteride), each 4 years in duration, there was no evidence of increased sexual adverse reactions…with increased duration of treatment” (GlaxoSmithKline, 2014a).

Our recent meta-analysis of reports of clinical trials of finasteride for androgenic alopecia found that adverse event reporting was of poor quality, systematically biased, not generalizable to routine practice, and that most subjects had ≤1 year of finasteride exposure (Belknap et al., 2015). Fifteen systematic reviews or meta-analyses of 5α-RI clinical trial reports each concluded that 5α-RI-associated sexual adverse events are infrequent, mild, and reversible. None of these prior meta-analyses assessed the adequacy of evaluation of adverse events in primary clinical trial reports (Boyle, Gould & Roehrborn, 1996; Chin, 2013; Edwards & Moore, 2002; Gacci et al., 2014; Gupta & Charrette, 2014; Jimenez Cruz, Quecedo Gutierrez & Del Llano Senaris, 2003; Mella et al., 2010; Oelke et al., 2015; Park & Choi, 2014; Tacklind et al., 2010; Trost, 2013; Varothai & Bergfeld, 2014; Wu et al., 2014; Yin et al., 2015; Yuan et al., 2015). A meta-analysis of clinical trials in men with prostatic hyperplasia found that the risk of ED was significantly increased with combined 5α-RI plus α_2_ adrenergic receptor blocker compared to α_2_ adrenergic receptor blocker alone (Favilla et al., 2016). However, an observational study found that the risk of ED was not increased with combined 5α-RI plus α_2_ adrenergic receptor blocker compared to α_2_ adrenergic receptor blocker alone (Hagberg et al., 2016). A case-control study found that sexual and erectile function of men exposed to finasteride 1 mg daily did not differ from controls (Tosti, Piraccini & Soli, 2001), and an observational study found that sexual function in men did not decline over the first 4–6 months of exposure to finasteride 1 mg (Tosti et al., 2004). Some commentators still assert that 5α-RIs are safe (Hirshburg et al., 2016; Mondaini et al., 2007; Singh & Avram, 2014).

There is limited information available about the effects of prolonged 5α-RI exposure on risk of persistent erectile dysfunction (PED). A 4-year study found that the rate of severe sexual adverse events was similar in subjects randomized to finasteride or placebo and that the rate of persistence of sexual dysfunction in finasteride subjects did not significantly differ from that in placebo subjects. However, this study relied on spontaneous reports of subjects for detection of sexual adverse events and did not use a validated measure of sexual function (Wessells et al., 2003). A report of a 5-year study found that about 10% of men with prostatic hyperplasia experienced erectile dysfunction (ED) with finasteride exposure but this report omitted mention of persistence of ED (Hudson et al., 1999). Another long-term study found a progressive and sustained worsening of erectile function in men with prostatic hyperplasia who had continued exposure to finasteride, with a clinically significant decline of six to eight points in international index of erectile function (IIEF)-EF score over 45 months (Traish et al., 2015a).

There are a disproportionate number of spontaneous reports of finasteride-associated persistent sexual dysfunction in the FDA’s adverse event reporting system (Ali, Heran & Etminan, 2015; Guo et al., 2016). There are also reports from uncontrolled case series of PED, low libido, loss of penile sensitivity, and lowered testosterone levels in young men that did not resolve after stopping finasteride (Chiriaco et al., 2016; Irwig, 2012a, 2012b, 2014; Irwig & Kolukula, 2011). Some reports describe men with symptoms beginning within days of initiating finasteride and persisting for years after stopping finasteride (Traish et al., 2011). PED has also been reported to occur with dutasteride exposure (Tsunemi et al., 2016). A recent functional magnetic resonance imaging (fMRI) study found that men with sexual adverse effects that persisted despite stopping finasteride did not have systemic androgen deficiency but did have fMRI responses to erotic stimuli that were consistent with diminished sexual arousal and depression (Basaria et al., 2016).

Given uncertainty about the frequency and severity of 5α-RI-associated sexual dysfunction, we analyzed existing clinical data for a large cohort of men prescribed finasteride or dutasteride to identify predictors of new PED. We hypothesized that longer 5α-RI exposure duration would increase the risk of PED. In addition to new PED, we also analyzed this data to identify predictors of new ED and new low libido.

## Methods

This is a stratified, multivariable quasi-experimental cohort study of sexual dysfunction in men exposed to 5α-RIs. A cohort of men, all of whom had 5α-RI exposure, was evaluated to determine the variables that predict the occurrence of new ED, new low libido, and new PED. Those men with shorter 5α-RI exposure served as a comparison control group for men with longer 5α-RI exposure. Variables included subject attributes, diseases, and drug exposures associated with sexual dysfunction. We used existing data from electronic medical records and a single-group study design. We also performed a naïve analysis comparing men with and without exposure to 5α-RIs. Our data source was the Northwestern Medicine Enterprise Data Warehouse (NMEDW), an electronic medical record (EMR) data repository for patients of Northwestern Medicine. The Northwestern University Institutional Review Board granted approval to conduct this research and granted a waiver of informed consent (Approval reference STU00037913). The clinicians providing medical care to these subjects and the subjects themselves were unaware of this study of existing data. Eligible subjects for evaluation for new ED and new low libido were men 16–89 years old with at least one clinical encounter and one diagnosis from January 1992 to September 2015. Eligible subjects for evaluation for new PED were men 16–89 years old with at least one clinical encounter and one diagnosis from January 1992 to September 2013.

### Exposure to 5α-RIs and other drugs

We used medication history and e-prescriptions to identify men prescribed 5α-RIs, non-steroidal anti-inflammatory drugs (NSAIDs), diuretics, antidepressants, acyclovir-type antivirals, and phosphodiesterase-5 inhibitors (PDE_5_Is), including combination forms (e.g., dutasteride + tamsulosin). Medication history was typically recorded by a mid-level practitioner with independent confirmation by a physician or other prescriber and is considered reliable at this institution. E-prescribing began in 2010; e-prescriptions were entered directly by prescribers. Prescription data included prescription dates, drug name, dose, and days of supply. Exposure duration was calculated as days from initial 5α-RI prescription to either onset of the adverse effect or last appearance in dataset, with omission of duplicate prescriptions and exclusion of intervals without documented 5α-RI exposure. The analysis included separate variables for finasteride ≤1.25 mg vs finasteride 5 mg; dutasteride vs finasteride; and finasteride ≤1.25 mg vs (finasteride >1.25 mg or dutasteride at any dose). We classified finasteride dosing as either ≤1.25 mg or >1.25 mg because tablet splitting of the 5 mg finasteride oral solid dosage form was commonly used to lower costs when prescribed for androgenic alopecia. For drugs approved after January 1992, assessment for drug exposure began with date of FDA approval for marketing.

### Diagnoses, adverse effects, and other attributes

We used the term “impotence” for the database searches because this is the target term to which synonyms are mapped in international classification of diseases codes (ICD-9), and was thereby the term used for encoding structured data on diagnosis into the EMR that was our primary data source. The International Conference for the Ninth Revision of the International Classification of Diseases met in 1975; final proposals for ICD-9 were ratified in 1978. Thus, the terminology for ICD-9 reflects that in use in the late 1970s. The term “impotence” is now deprecated in medical parlance and has been replaced with the term “erectile dysfunction.” “Male erectile dysfunction” is the term used in ICD-10. We used the term “prostate disease” to aggregate ICD-9 terms for hyperplasia of the prostate, including with or without urinary obstruction, and with or without other urinary symptoms.

For each subject, we identified physician-determined diagnosis of impotence (ED), low libido, alopecia, prostate disease, prostate cancer, prostate surgery, Peyronie’s disease, cardiovascular disease, hypertension, diabetes mellitus, obesity, alcoholism, tobacco use, depression, herpes simplex virus (HSV-1 or HSV-2) infection, and HIV infection using ICD-9. Surgical procedures were encoded using ICD-9 procedure codes, AMA current procedural terminology (CPT) codes, and institution-specific billing codes. For manual review of narratives, impotence (ED) was defined as “inability to initiate and maintain erection sufficient for sexual intercourse.” We calculated body mass index from measured weights (kg) and heights (m). Laboratory data included glycosylated hemoglobin A_1c_, low-density lipoprotein, triglyceride, and magnesium. We assessed extent of healthcare utilization as number of clinical encounters before onset of the adverse effect, and also between initial 5α-RI exposure and onset of the adverse effect.

The adverse effect of ED required both a physician-determined diagnosis of ED and a contemporaneous prescription for a PDE_5_I during 1998 or later—when sildenafil, the first PDE_5_I, became available. The adverse effect of low libido required a physician-determined diagnosis of decreased libido. Designation of either new ED or new low libido additionally required that there be no prior diagnosis of ED, nor of PDE_5_I use, nor of low libido before initial 5α-RI exposure. PED additionally required description by a physician in the clinical narrative of new ED lasting ≥90 days after stopping 5α-RI (per FDA criterion for PED; Kothary, Diak & McMahon, 2011), as determined by manual review; a second reviewer independently assessed the relevant text and rare differences between reviewers were reconciled by consensus. The date of resolution of ED was that reported by the subject and recorded by the physician in the clinical narrative or the first encounter where the physician documented resolution, or the last encounter where the physician recorded that the subject continued to experience ED. Thus, PED required the simultaneous presence of a new diagnosis of an adverse effect (ED), discontinuation of the suspect drug (5α-RI), new use of an antidote (PDE_5_I), and documented persistence of the adverse effect after stopping the suspect drug (5α-RI). This case definition of 5α-RI-associated PED is analogous to trigger tools that have demonstrated high reliability in drug safety studies (Classen et al., 2011; Naessens et al., 2009; Resar, Rozich & Classen, 2003).

### Definition of cohorts of men without prior sexual dysfunction and exposed to a 5α-RI

For the cohort of all men, we identified men prescribed a 5α-RI with no recorded diagnosis of ED nor of low libido nor a record of PDE_5_I use prior to initial prescription of a 5α-RI. For the cohort of young men, we identified men with 5α-RI exposure who were 16–42 years old, had exposure to finasteride ≤1.25 mg/day, had neither exposure to finasteride >1.25 mg/day nor exposure to dutasteride, nor prostate surgery, nor ED, nor low libido, nor PDE_5_I use prior to finasteride exposure.

### Statistical analysis

We used a single-group study design for the main analysis. The source data did not satisfy the assumptions underlying analytical methods that are based on the general linear model or maximum likelihood function (Grimm & Yarnold, 1995, 2000). Accordingly, all analyses used optimal discriminant analysis, an exact, non-parametric statistical method (Arozullah et al., 2003; Belknap et al., 2008; Nebeker et al., 2007; Smart et al., 2008) to model ED, low libido, and PED. Use of these non-parametric methods simplifies and standardizes presentation and interpretation of statistical findings, avoids certain ambiguities that arise with alternative multivariable analytic methods, ensures valid *p*-values, and also identifies models that maximize predictive accuracy (i.e., as opposed to models that maximize explained variation or that maximize the value of the likelihood function; Grimm & Yarnold, 1995, 2000; Linden & Yarnold, 2016a, 2016b, 2016c, 2016d, 2016e; Linden, Yarnold & Nallamothu, 2016; Yarnold & Soltysik, 2005). All calculations were either computed exactly or were estimated using Monte Carlo simulation. Univariable analyses were conducted using optimal data analysis (ODA) software (Yarnold & Soltysik, 2005), and multivariable analyses using classification tree analysis (CTA) software (Ostrander et al., 1998; Yarnold & Soltysik, 2016; Yarnold, Soltysik & Bennett, 1997). These analyses identify the model that explicitly maximizes predictive accuracy as indexed by the effect strength for sensitivity (ESS) statistic—a chance-corrected and maximum-corrected measure of classification accuracy for which 0 is the discrimination accuracy expected by chance and 100 is perfect intergroup discrimination (Linden & Yarnold, 2016c; Yarnold, Soltysik & Bennett, 1997). As ODA analyses require no distributional assumptions about the data, permutation probability is used to compute statistical significance as exact *p*-values. Where multiple statistical hypotheses were tested, the Šidák multiple comparisons method was used to ensure the statistical reliability at the experimentwise (*p* ≤ Šidák criterion) or the generalized (per-comparison *p* ≤ 0.05) criterion (Yarnold & Soltysik, 2005, 2016).

Results for univariable analyses of the relationship between adverse effects and exposure variables are presented in descending order by ESS. The multivariable relationship between adverse effects and exposure variables was modeled using hierarchically optimal CTA, an algorithm that chains ODA analyses over all strata and over each branch of the classification tree to explicitly maximize ESS for the overall model. As with ODA, CTA analyses also require no distributional assumptions about the data, so permutation probability is used to compute statistical significance as exact *p*-values (Yarnold & Soltysik, 2016; Yarnold, Soltysik & Martin, 1994). Multivariable models identified by CTA drew potential predictors from a pool of demographic variables, subject attributes, healthcare utilization measures, disease classifications, and drug exposures (Table 3). Multivariable model endpoints were constrained a priori to be at least 10% of overall cohort size to insure adequate statistical power as well as to inhibit overfitting (i.e., identifying strata with insufficiently large sample size) and thereby improve reproducibility of the findings (Linden & Yarnold, 2016c; Yarnold & Soltysik, 2016; Yarnold, Soltysik & Martin, 1994). Under this minimum sample size constraint, the CTA algorithm uses a search procedure that explicitly assures that the reported classification tree achieves greater accuracy than any other possible alternative classification tree. We prospectively validated the CTA models of ED and low libido in the subcohort of 5α-RI exposed men who had no identified sexual dysfunction during the main study interval by using data from the 6-month interval immediately following the end of the main study interval.

## Results

### Demographics and naïve analysis in exposed vs non-exposed men

The repository contained medical records for 691,268 men (Table 1) of whom 17,475 (2.5%) had 5α-RI exposure; of these, 15,634 (89.5%) had no prior diagnosis of ED nor of low libido, nor of PDE_5_I use. Men exposed to 5α-RIs were more likely than unexposed men to have the diagnosis of ED (number needed to harm (NNH) 17.3, ESS 6.4%, *p* < 0.0001) and of low libido (NNH 73.5, ESS 4.0%, *p* < 0.0001) and to have been prescribed a PDE_5_I (NNH 10.6, ESS 5.8%, *p* < 0.0001) (Table 2A). There were 327,437 men 16–42 years old, with 743 exposed either to finasteride >1.25 mg/day or to dutasteride, and 5,582 (1.7%) exposed to finasteride ≤1.25 mg/day. Compared to young men without 5α-RI exposure, those young men with exposure to finasteride ≤1.25 mg/day were more likely to have the adverse effect of ED (NNH 31.1, ESS 6.7%, *p* < 0.0001) and of low libido (NNH 51.0, ESS 7.2%, *p* < 0.0001) (Table 2B). Among 16,032 men with prostatic hyperplasia and with an encounter and a diagnosis recorded during 2014 (the last complete year in the dataset), 3,890 (24.3%) had 5α-RI exposure.

**Table 1:** Baseline characteristics of cohort members^a^ (total *N* = 691,268). Exposed to 5α-RI drugs vs unexposed. Men exposed to 5α-RIs differed from men unexposed to 5α-RIs for several characteristics relevant to the frequency or to the detection of sexual dysfunction. Notably, exposed men had more years in the cohort (i.e., longer duration of time represented in the medical record). Men exposed to finasteride ≤1.25 mg daily were younger and were less likely to have prostate disease than men exposed to finasteride >1.25 mg daily or exposed to dutasteride.

Characteristic	Cohort														
	Unexposed (*N* = 673,793)			Finasteride ≤1.25 mg (*N* = 7,419)			Finasteride >1.25 mg (*N* = 7,187)			Dutasteride (*N* = 1,921)			All exposed (*N* = 17,475)		
Mean year of cohort entry	2005 ± 6.00			2006 ± 5.42			2004 ± 6.93			2005 ± 7.04			2005 ± 6.42		
Median year of cohort entry	2006			2006			2004			2007			2005		
Median age at cohort entry	38.9			30.4			63.8			64.3			51.0		
IQR of age at cohort entry	28.7–53.6			26.2–36.8			53.6–71.5			56.2–72.4			30.8–66.3		
Median years in cohort (IQR)	0.9 (0.0–5.6)			6.1 (2.1–11.3)			8.5 (2.2–14.6)			5.4 (1.2–13.0)			7.1 (2.1–13.1)		
**Attribute**	***N***	**%**		***N***	**%**		***N***	**%**		***N***	**%**		***N***	**%**	
Use of prescription NSAID^b^	180,375	26.8		2,604	35.1		5,037	70.1		1,346	70.1		9,759	55.8	
Hypertension^c^	117,585	17.5		945	12.7		4,285	59.6		1,035	53.9		6,942	39.7	
Smoking^c^	111,798	16.6		1,336	18.0		3,033	42.2		798	41.5		5,663	32.4	
Vascular disease^c^	88,752	13.2		538	7.3		3,644	50.7		835	43.5		5,623	32.2	
Use of diuretic drug^b^	60,680	9.0		418	5.6		3,074	42.8		726	37.8		4,696	26.9	
Diabetes mellitus^c^	45,431	6.7		150	2.0		1,599	22.2		352	18.3		2,348	13.4	
Use of SSRI drug^b^	35,570	5.3		966	13.0		1,183	16.5		269	14.0		2,614	15.0	
Depression^c^	34,586	5.1		673	9.1		1,070	14.9		195	10.2		2,101	12.0	
Diagnosis of alcoholism^c^	31,310	4.6		186	2.5		264	3.7		36	1.9		535	3.1	
Diagnosis of obesity^c^	30,069	4.5		365	4.9		797	11.1		188	9.8		1,504	8.6	
Prostate disease^c^	24,936	3.7		181	2.4		4,438	61.8		940	48.9		6,349	36.3	
Prostate cancer^c^	18,207	2.7		57	0.8		770	10.7		187	9.7		1,155	6.6	
Use of cyclovir drug^b^	17,850	2.6		673	9.1		591	8.2		113	5.9		1,468	8.4	
History of prostate surgery^d^	13,096	1.9		65	0.9		1,428	19.9		291	15.1		2,073	11.9	
HSV^c^	6,647	1.0		327	4.4		169	2.4		25	1.3		543	3.1	
Alopecia^c^	2,938	0.4		3,078	41.5		457	6.4		9	0.5		3,574	20.5	
Peyronie’s disease^c^	840	0.1		41	0.6		47	0.7		18	0.9		111	0.6	
	**Unexposed**			**Finasteride ≤1.25 mg**			**Finasteride >1.25 mg**			**Dutasteride**			**All exposed**		
**Attribute**^**e**^	***N***	**Median**	**IQR**	***N***	**Median**	**IQR**	***N***	**Median**	**IQR**	***N***	**Median**	**IQR**	***N***	**Median**	**IQR**
Body mass index	335,057	26.9	24.3–30.4	6,503	25.8	24.0–28.1	6,762	26.9	24.4–30.2	1,732	27.6	24.9–30.8	15,933	26.4	24.3–29.3
Triglycerides	177,526	100.0	69.0–149.0	4,535	88.0	63.0–127.0	4,057	95.5	68.0–136.0	792	99.0	73.0–141.1	10,009	92.0	66.0–132.0
LDL cholesterol	154,340	107.0	85.0–129.5	4,384	112.0	93.0–132.0	3,722	88.0	69.0–112.0	737	90.0	70.0–113.0	9,437	100.0	78.0–123.0
Magnesium	117,488	2.0	1.9–2.1	763	2.0	1.9–2.2	3,677	2.0	1.9–2.1	741	2.0	1.9–2.1	5,824	2.0	1.9–2.1
Hemoglobin A_1c_	48,722	5.6	5.3–6.4	786	5.3	5.1–5.6	1,862	5.8	5.5–6.5	412	5.8	5.4–6.5	3,396	5.7	5.3–6.3

**Notes:**

a Selection criteria for cohort: all men with at least one diagnosis in the medical record and between 16 and 89 years old as of their last encounter.

b Based on prescription data in the electronic medical record.

c Based on ICD-9 codes in the electronic medical record.

d Based on ICD-9 procedure codes, AMA current procedural terminology (CPT) codes, and institution-specific billing codes.

e Value for each individual is the median of all values in the electronic medical record.

**Table 2:** Naïve analysis men exposed vs unexposed to 5α-reductase inhibitors. (A). Men exposed to 5α-RIs had a higher risk of erectile dysfunction (based solely on ICD-9 code), PDE_5_I use, and low libido in the cohort of all men. The higher risk was found for all men exposed to 5α-RIs vs unexposed, men exposed to either finasteride >1.25 mg daily or to dutasteride vs unexposed, finasteride >1.25 daily vs unexposed, finasteride ≤1.25 mg vs unexposed, or dutasteride vs unexposed. (B). Men younger than 42 exposed to finasteride ≤1.25 mg/day had a higher risk of erectile dysfunction (based on ICD-9 code alone), PDE_5_I use, and low libido compared with unexposed men.

	Effect variable	*N* (exposure/effect)			NNH	Risk ratio	Specificity (%)	Sensitivity (%)	NPV (%)	PPV (%)	ESS (%)	*p* value
		+/+	+/−	−/+								
**A. Univariate predictors for all men (*N* = 691,268): cohorts exposed to 5α-RI drugs compared to unexposed cohort**												
*Drug exposure*												
All 5α-RIs exposed (*N* = 17,475) vs unexposed (*N* = 673,793)	Erectile dysfunction^a^	1,381	16,094	14,367	17.3	3.706	97.6	8.8	97.9	7.9	6.4	<0.0001
	PDE-5 inhibitor^b^	2,346	15,129	26,823	10.6	3.372	97.7	8.0	96.0	13.4	5.8	<0.0001
	Low libido^c^	381	17,094	5,520	73.5	2.661	97.5	6.5	99.2	2.2	4.0	<0.0001
High-dose 5α-RIs exposed (*N* = 9363) vs unexposed (*N* = 673,793)	Erectile dysfunction^a^	915	8,448	14,367	13.1	4.583	98.7	6.0	97.9	9.8	4.7	<0.0001
	PDE-5 inhibitor^b^	1,506	7,857	26,823	8.3	4.040	98.8	5.3	96.0	16.1	4.1	<0.0001
	Low libido^c^	182	9,181	5,520	88.9	2.373	98.6	3.2	99.2	1.9	1.8	<0.0001
High-dose finasteride exposed (*N* = 6,547) vs unexposed (*N* = 673,793)	Erectile dysfunction^a^	628	5,919	14,367	13.4	4.499	99.1	4.2	97.9	9.6	3.3	<0.0001
	PDE-5 inhibitor^b^	1,027	5,520	26,823	8.5	3.940	99.2	3.7	96.0	15.7	2.8	<0.0001
	Low libido^c^	123	6,424	5,520	94.4	2.293	99.0	2.2	99.2	1.9	1.2	<0.0001
Low-dose finasteride exposed (*N* = 7,419) vs unexposed (*N* = 673,793)	Erectile dysfunction^a^	383	7,036	14,367	33.0	2.421	98.9	2.6	97.9	5.2	1.5	<0.0001
	PDE-5 inhibitor^b^	707	6,712	26,823	18.0	2.394	99.0	2.6	96.0	9.5	1.5	<0.0001
	Low libido^c^	175	7,244	5,520	65.0	2.879	98.9	3.1	99.2	2.4	2.0	<0.0001
Dutasteride exposed (*N* = 1,921) vs unexposed (*N* = 673,793)	Erectile dysfunction^a^	162	1,759	14,367	15.9	3.955	99.7	1.1	97.9	8.4	0.9	<0.0001
	PDE-5 inhibitor^b^	280	1,641	26,823	9.4	3.661	99.7	1.0	96.0	14.6	0.8	<0.0001
	Low libido^c^	37	1,884	5,520	90.3	2.351	99.7	0.7	99.2	1.9	0.4	<0.0001
**B. Univariate predictors for men <42^d^ (*N* = 326,694): cohort exposed to low-dose finasteride (≤1.25 mg/day), compared to unexposed cohort**												
*Drug exposure*												
Low-dose finasteride exposed (*N* = 5,582) vs unexposed (*N* = 321,112)	Erectile dysfunction^a^	222	5,360	2,447	31.1	5.219	98.3	8.3	99.2	4.0	6.7	<0.0001
	PDE-5 inhibitor^b^	386	5,196	3,787	17.4	5.864	98.4	9.2	98.8	6.9	7.6	<0.0001
	Low libido^c^	133	5,449	1,359	51.0	5.630	98.3	8.9	99.6	2.4	7.2	<0.0001

**Notes:**

NNH, Number Needed to Harm = 1/attributable risk; NPV, Negative Predictive Value; PPV, Positive Predictive Value; ESS, Effect Strength for Sensitivity (defined in text).

a Adverse event of erectile dysfunction is the earliest occurrence of ICD-9 code 607.84 or v41.7 after exposure to 5α-RI with a concurrent prescription for a PDE_5_I, and not present prior to exposure.

b Based on prescription dates in the electronic medical record.

c Based on ICD-9 codes in the electronic medical record.

d Men <42: Males between 16 and 42 years old (at time of first exposure to any 5-αRI), either not exposed to any 5-α reductase inhibitor or exposed to low-dose finasteride, excluding high-dose 5α-RIs.

### New erectile dysfunction and new low libido in men exposed to a 5α-RI

Among the 15,634 men with 5α-RI exposure and without prior sexual dysfunction, 699 (4.5%) developed new ED and 210 (1.3%) developed new low libido.

#### Univariable predictors for new erectile dysfunction and new low libido in all men exposed

5α-Reductase inhibitor exposure duration was a significant predictor of new ED (cutpoint >90.5 days of 5α-RI exposure, NNH 29.7, ESS 19.6%, *p* < 0.0001). Of the 29 significant predictors of new ED, four were more accurate predictors than 5α-RI exposure duration: prostate disease, prostate surgery, number of encounters, and number of encounters after 5α-RI exposure (Table 3A). Of the 15 significant predictors of new low libido, 5α-RI exposure duration was the most accurate predictor (cutpoint >96.5 days of 5α-RI exposure, NNH 76, ESS 24.8%, *p* < 0.0001) (Table 3A).

**Table 3:** Univariate risk factors for new erectile dysfunction, new low libido, and new persistent erectile dysfunction. (A). For men exposed to 5α-RIs, there were 29 statistically significant risk factors (*p* < 0.05) predicting new erectile dysfunction after exposure to 5α-RIs. Number of days of 5α-RI exposure was the fifth most important risk factor for new erectile dysfunction. Men with >90.5 days of 5α-RI exposure had a 2.2-fold higher risk of new erectile dysfunction compared with men with ≤90.5 days of 5α-RI exposure. There were nine statistically significant risk factors (*p* < 0.05) predicting new low libido after exposure to 5α-RIs. Number of days of 5α-RI exposure was the most important risk factor for new low libido. Men with >96.5 days of 5α-RI exposure had a three-fold higher risk of new low libido compared with men with ≤96.5 days of 5α-RI exposure. (B). For men exposed to 5α-RIs, there were 26 statistically significant risk factors (*p* < 0.05) predicting new persistent erectile dysfunction after exposure to 5α-RIs. Number of days of 5α-RI exposure was the third most important risk factor for new persistent erectile dysfunction. Men with >179.5 days of 5α-RI exposure had a 2.3-fold higher risk of new persistent erectile dysfunction compared with men with ≤179.5 days of 5α-RI exposure. For men younger than 42 years and exposed to 5α-RIs, there were nine statistically significant risk factors (*p* < 0.05) predicting new persistent erectile dysfunction after exposure to 5α-RIs. Number of days of 5α-RI exposure was the most important risk factor for new persistent erectile dysfunction. Men with >205 days of 5α-RI exposure had a 4.9-fold higher risk of new erectile dysfunction compared with men with ≤205 days of 5α-RI exposure.

RIsk factor	*N* (exposure/effect)			NNH	Risk ratio	Specificity (%)	Sensitivity (%)	NPV (%)	PPV (%)	ESS (%)	*p* value
	+/+	+/−	−/+								
**A. (i) Univariable predictors of new erectile dysfunction^a^ in men prescribed 5α-reductase inhibitors^c^ 699 of 15,634 (4.5%)**											
Prostate disease^d^	403	4,792	296	20.3	2.7	67.9	57.7	97.2	7.8	25.6	<0.0001
Number of encounters >27.5	506	7,312	193	25.0	2.6	51.0	72.4	97.5	6.5	23.4	<0.0001
Encounters after 5α-RI exposure >12.5^e^	534	8,129	165	26.3	2.6	45.6	76.4	97.6	6.2	22.0	<0.0001
Prostate surgery^f^	203	1,300	496	10.0	3.8	91.3	29.0	96.5	13.5	20.3	<0.0001
Days of exposure to 5α-RI >90.5^e^	497	7,685	202	29.7	2.2	48.5	71.1	97.3	6.1	19.6	<0.0001
Encounters prior to 5α-RI exposure >8.5^e^	426	6,174	273	29.1	2.1	58.7	60.9	97.0	6.5	19.6	<0.0001
Prescription NSAID^g^	489	7,953	210	34.8	2.0	46.7	70.0	97.1	5.8	16.7	<0.0001
Age ≤72.6^h^	608	1,0544	91	29.2	2.7	29.4	87.0	98.0	5.5	16.4	<0.0001
Hypertension^d^	371	5,507	328	33.9	1.9	63.1	53.1	96.6	6.3	16.2	<0.0001
Depression^d^	162	1,591	537	18.6	2.4	89.3	23.2	96.1	9.2	12.5	<0.0001
Number of progress notes >8.5	343	5,470	356	43.9	1.6	63.4	49.1	96.4	5.9	12.4	<0.0001
High-dose finasteride (>1.25 mg/day)^g^	393	6,557	306	46.9	1.6	56.1	56.2	96.5	5.7	12.3	<0.0001
Progress notes after 5α-RI exposure >2.5^e^	473	8,346	226	48.8	1.6	44.1	67.7	96.7	5.4	11.8	<0.0001
Smoking^d^	284	4,561	415	49.6	1.5	69.5	40.6	96.2	5.9	10.1	<0.0001
Prostate cancer^d^	108	816	591	13.0	2.9	94.5	15.5	96.0	11.7	10.0	<0.0001
Androgen drug^g^	64	229	635	5.6	5.3	98.5	9.2	95.9	21.8	7.6	<0.0001
Diagnosis of obesity^d^	104	1,140	595	23.7	2.0	92.4	14.9	95.9	8.4	7.2	<0.0001
Vascular disease^d^	260	4,500	439	70.2	1.4	69.9	37.2	96.0	5.5	7.1	<0.0001
SSRI drug^g^	147	2,106	552	41.7	1.6	85.9	21.0	95.9	6.5	6.9	<0.0001
Body mass index >27.8^i^	298	4,863	396	72.5	1.3	63.9	42.9	95.6	5.8	6.9	0.0038
Cyclovir drug^g^	100	1,140	599	25.6	1.9	92.4	14.3	95.8	8.1	6.7	<0.0001
Triglyceride level >71.8^i^	420	5,553	140	54.6	1.4	31.5	75.0	94.8	7.0	6.5	0.0176
Diuretic drug^g^	210	3,818	489	100.0	1.2	74.4	30.0	95.8	5.2	4.5	0.0097
Dutasteride^g^	137	2,333	562	78.3	1.3	84.4	19.6	95.7	5.5	4.0	0.0052
History of herpes infection^d^	47	412	652	16.8	2.4	97.2	6.7	95.7	10.2	4.0	<0.0001
Diabetes mellitus^d^	113	1,852	586	68.3	1.3	87.6	16.2	95.7	5.8	3.8	0.0030
Anti-androgen drug^g^	22	208	677	19.3	2.2	98.6	3.1	95.6	9.6	1.8	0.0005
Peyronie’s disease^d^	10	55	689	9.1	3.5	99.6	1.4	95.6	15.4	1.1	0.0007
**(ii) Univariable predictors of new low libido^b^ in men prescribed 5α-reductase inhibitors^c^ 210 of 15,634 (1.34%)**											
HIV^d^	13	145	686	26.3	1.9	99.0	1.9	95.6	8.2	0.9	0.0313
Days of exposure to 5α-RI >96.5^e^	161	8,006	49	76.0	3.0	48.1	76.7	99.3	2.0	24.8	<0.0001
Age ≤67.4^h^	179	9,361	31	73.1	3.7	39.3	85.2	99.5	1.9	24.6	<0.0001
Encounters after 5α-RI exposure >9.5^e^	170	9,532	40	92.8	2.6	38.2	81.0	99.3	1.8	19.1	<0.0001
Number of encounters >34.5	133	6,871	77	99.3	2.1	55.5	63.3	99.1	1.9	18.8	<0.0001
SSRI drug^g^	68	2,185	142	51.1	2.8	85.8	32.4	98.9	3.0	18.2	<0.0001
Androgen drug^g^	39	254	171	8.2	11.9	98.4	18.6	98.9	13.3	16.9	<0.0001
Depression^d^	58	1,695	152	45.2	3.0	89.0	27.6	98.9	3.3	16.6	<0.0001
Prescription NSAID^g^	139	8,303	71	151.7	1.7	46.2	66.2	99.0	1.6	12.4	0.0002
LDL cholesterol >106.8^i^	96	3,432	85	113.2	1.5	56.9	53.0	98.2	2.7	10.0	0.0438
Encounters prior to 5α-RI exposure >11.5^e^	98	5677	112	178.3	1.5	63.2	46.7	98.9	1.7	9.9	0.0192
Diagnosis of obesity^d^	34	1,210	176	66.2	2.2	92.2	16.2	98.8	2.7	8.3	0.0002
Cyclovir drug^g^	33	1,207	177	69.9	2.2	92.2	15.7	98.8	2.7	7.9	0.0003
History of herpes infection^d^	18	441	192	37.6	3.1	97.1	8.6	98.7	3.9	5.7	<0.0001
Alcohol abuse^d^	15	422	195	46.5	2.7	97.3	7.1	98.7	3.4	4.4	0.0012
Peyronie’s disease^d^	5	60	205	15.7	5.8	99.6	2.4	98.7	7.7	2.0	0.0020
**B. Univariable predictors of persistent erectile dysfunction^j^ in men prescribed 5α-reductase inhibitors^k^ 167 of 11,909 (1.40%)**											
Prostate surgery^f^	66	120	101	2.9	41.2	99.0	39.5	99.1	35.5	38.5	<0.0001
Prostate disease^d^	103	3,950	64	57.9	3.1	66.4	61.7	99.2	2.5	28.0	<0.0001
5α-RI exposure >179.5 days^e^	113	5,555	54	88.6	2.3	52.7	67.7	99.1	2.0	20.4	<0.0001
Prescription NSAID^g^	121	6,173	46	90.6	2.3	47.4	72.5	99.2	1.9	19.9	<0.0001
Hypertension^d^	94	4,392	73	89.9	2.1	62.6	56.3	99.0	2.1	18.9	<0.0001
Age ≤71.8^h^	148	8,212	19	81.0	3.3	30.1	88.6	99.5	1.8	18.7	<0.0001
Encounters prior to 5α-RI exposure >2.5^e^	128	6,842	39	95.5	2.3	41.7	76.6	99.2	1.8	18.4	<0.0001
Age at earliest 5α-RI exposure ≤70.3^h^	150	8,509	17	82.7	3.3	27.5	89.8	99.5	1.7	17.4	0.0004
Number of encounters >13.5	125	6,758	42	102.0	2.2	42.4	74.9	99.2	1.8	17.3	<0.0001
Prostate cancer^d^	37	739	130	27.8	4.1	93.7	22.2	98.8	4.8	15.9	<0.0001
Any high-dose 5α-reductase inhibitor^g^	114	6,400	53	130.3	1.8	45.5	68.3	99.0	1.8	13.8	0.0004
High-dose finasteride (>1.25 mg/day)^g^	91	5,047	76	154.2	1.6	57.0	54.5	98.9	1.8	11.5	0.0030
Encounters after 5α-RI exposure >3.5^e^	140	8,522	27	127.4	1.9	27.4	83.8	99.2	1.6	11.3	0.0162
Smoking^d^	67	3,464	100	142.1	1.6	70.5	40.1	98.8	1.9	10.6	0.0042
Androgen drug^g^	20	220	147	14.1	6.6	98.1	12.0	98.7	8.3	10.1	<0.0001
Depression^d^	34	1,239	133	70.4	2.1	89.4	20.4	98.7	2.7	9.8	<0.0001
SSRI drug^g^	39	1,618	128	90.5	1.9	86.2	23.4	98.8	2.4	9.6	0.0004
Dutasteride^g^	40	1,902	127	127.3	1.6	83.8	24.0	98.7	2.1	7.8	0.0096
Vascular disease^d^	66	3,765	101	211.6	1.4	67.9	39.5	98.7	1.7	7.5	0.0487
Diuretic drug^g^	55	2,998	112	186.3	1.4	74.5	32.9	98.7	1.8	7.4	0.0340
Diabetes mellitus^d^	31	1,429	136	121.7	1.6	87.8	18.6	98.7	2.1	6.4	0.0121
Cyclovir drug^g^	20	865	147	107.9	1.7	92.6	12.0	98.7	2.3	4.6	0.0294
Diagnosis of obesity^d^	19	827	148	110.1	1.7	93.0	11.4	98.7	2.2	4.3	0.0333
Alcohol abuse^d^	10	307	157	55.5	2.3	97.4	6.0	98.6	3.2	3.4	0.0142
HIV^d^	7	128	160	26.1	3.8	98.9	4.2	98.6	5.2	3.1	0.0035
Anti-androgen drug^g^	7	168	160	37.9	2.9	98.6	4.2	98.6	4.0	2.8	0.0103
**C. Univariable risk factors for persistent erectile dysfunction^j^ in men <42 years old exposed to finasteride 1.25 mg/day^l^ 34 of 4,284 (0.79%)**											
Finasteride exposure >205.0 days^e^	30	2,557	4	108.2	4.9	39.8	88.2	99.8	1.2	28.1	0.0039
Cyclovir drug^g^	12	325	22	33.3	6.4	92.4	35.3	99.4	3.6	27.6	<0.0001
SSRI drug^g^	13	478	21	47.8	4.8	88.8	38.2	99.4	2.6	27.0	<0.0001
Depression^d^	11	329	23	37.7	5.5	92.3	32.4	99.4	3.2	24.6	<0.0001
Prescription NSAID^g^	16	1,181	18	132.7	2.3	72.2	47.1	99.4	1.3	19.3	0.0180
Smoking^d^	9	582	25	118.2	2.2	86.3	26.5	99.3	1.5	12.8	0.0447
Hypertension^d^	7	340	27	75.1	2.9	92.0	20.6	99.3	2.0	12.6	0.0159
HIV^d^	4	38	30	11.3	13.5	99.1	11.8	99.3	9.5	10.9	0.0004
Diabetes mellitus^d^	2	39	32	24.2	6.5	99.1	5.9	99.2	4.9	5.0	0.0436

**Notes:**

Excludes risk factors with *p* ≥ 0.05.

NNH, Number Needed to Harm = 1/attributable risk; NPV, Negative Predictive Value; PPV, Positive Predictive Value; ESS, Effect Strength for Sensitivity (defined in text).

a The adverse effect of erectile dysfunction is defined as the earliest occurrence of ICD-9 code 607.84 or v41.7 with a concurrent prescription for any PDE-5 inhibitor drug, after exposure to 5α-RI and not present prior to exposure.

b New low libido is defined based on relevant ICD-9 codes present after exposure to 5-αRI drugs but not present prior to exposure.

c Selection criteria for the cohort: exposed to one or more 5α-RI drugs; no diagnoses of erectile dysfunction or low libido prior to 5α-RI exposure; no use of PDE-5 inhibitors prior to 5α-RI exposure.

d Based on the presence of relevant ICD-9 codes in the medical record, without regard to diagnosis date.

e Exposure is based on prescription dates in the electronic medical record.

f Excludes surgery performed after the earliest occurrence of erectile dysfunction.

g Prescription issued at any time in the medical record.

h Age as of earliest diagnosis of sexual dysfunction; or, if no dysfunction, then age at last encounter in the medical record.

i Median values over the course of the medical record.

j New persistent erectile dysfunction is defined as erectile dysfunction persisting at least 90 days after discontinuation of 5α-RI drugs, based on manual review of the electronic medical record.

k New persistent erectile dysfunction is defined as erectile dysfunction persisting at least 90 days after discontinuation of 5-αRI drugs, based on manual review of the medical record.

l Selection criteria for the cohort: exposed to finasteride with dosage ≤1.25 mg/day; not exposed to finasteride with dosage >1.25 mg/day; and not exposed to dutasteride; no diagnoses of erectile dysfunction or low libido prior to 5α-RI exposure; no use of PDE-5 inhibitors prior to 5α-RI exposure; no prostate disease, prostate surgery, or prostate cancer; and age <42 years at time of first prescription for finasteride.

#### Multivariable models for new erectile dysfunction and new low libido in all men exposed

**Figure 1:**
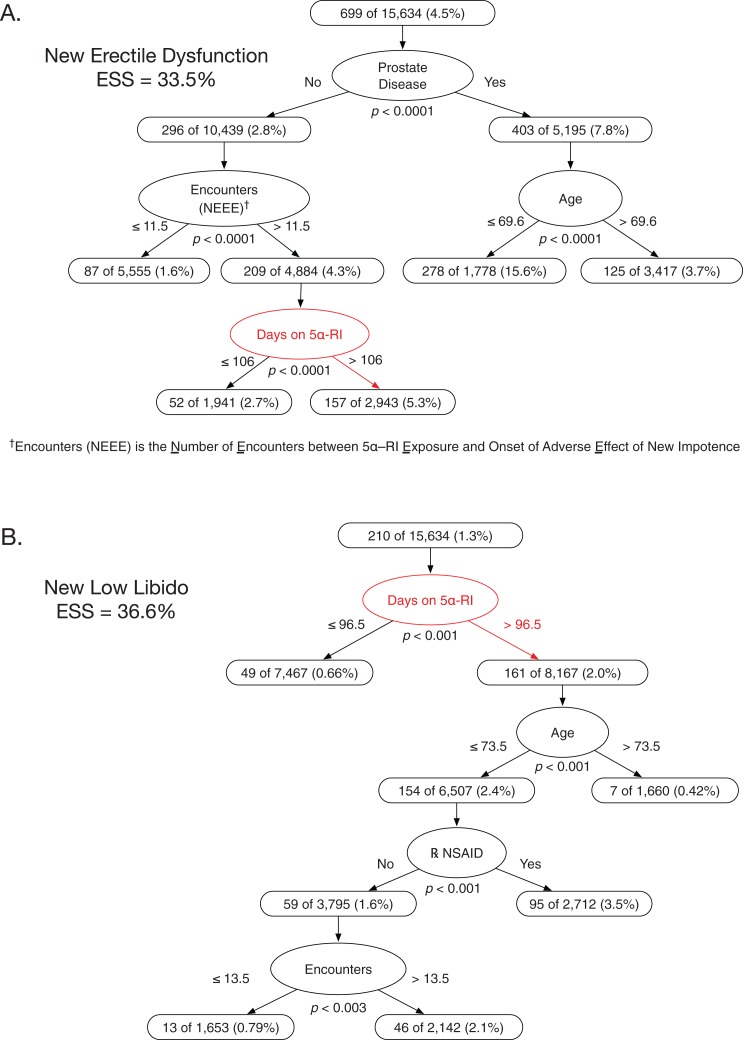
Classification tree analyses for erectile dysfunction and low libido in men prescribed 5α-reductase inhibitors. The two classification trees shown are those that predicted, respectively, new erectile dysfunction or new low libido after 5α-RI exposure with greater accuracy than any other possible alternative classification tree given the pool of exposure variables in Table 3. (A) This optimally predictive multivariable model for new erectile dysfunction had four variables: prostate disease, number of encounters between 5α-RI exposure and onset of impotence, age, and number of days on 5α-RIs. Men with no prostate disease, >11.5 clinical encounters, and >106 days of 5α-RI exposure had a 5.3% rate of new erectile dysfunction. (B) This optimally predictive multivariable model for new low libido had four variables: number of days of 5α-RI exposure, age, use of prescribed NSAIDs, and total number of clinical encounters. Men with >96.5 days of 5α-RI exposure, age ≤73.5 years, and use of NSAIDs had a 3.5% rate of new low libido.

The best multivariable model predicting new ED had four variables: prostate disease, number of encounters after initial 5α-RI exposure, 5α-RI exposure duration, and age (ESS 33.5%, all *p* ≤ 0.0001; prospective validity on 6-month holdout sample ESS 30.8%) (Fig. 1A). Among men without prostate disease and with > 11.5 encounters after 5α-RI exposure, the NNH for new ED was 37.7 for longer vs shorter 5α-RI exposure duration (cutpoint >106 days of 5α-RI exposure). The multivariable model predicting new low libido had four attributes: 5α-RI exposure duration, age, NSAID exposure (Y/N), and total number of clinical encounters (ESS 36.6%, all *p* ≤ 0.003; prospective validity on 6-month holdout sample ESS 34.2%) (Fig. 1B).

### New persistent erectile dysfunction in men exposed to a 5α-RI

Of the 11,909 men with 5α-RI exposure and without prior sexual dysfunction and who were evaluated for new PED, 167 (1.4%) developed new PED lasting for ≥90 days after stopping the 5α-RI (median 1,348 days after stopping 5α-RI, interquartile range (IQR) 631.5–2,320.5 days). Of the 530 men with new ED, 167 (31.5%) had new PED.

#### Univariable predictors for new persistent erectile dysfunction in all men exposed

5α-Reductase inhibitor exposure duration was the third most accurate predictor of PED (cutpoint >179.5 days of 5α-RI exposure, NNH 88.6, ESS 20.4%, *p* < 0.0001). Of the 26 statistically significant predictors of PED, only prostate surgery and prostate disease were more accurate predictors than 5α-RI exposure duration (Table 3B).

#### Multivariable model for new persistent erectile dysfunction in all men exposed

**Figure 2:**
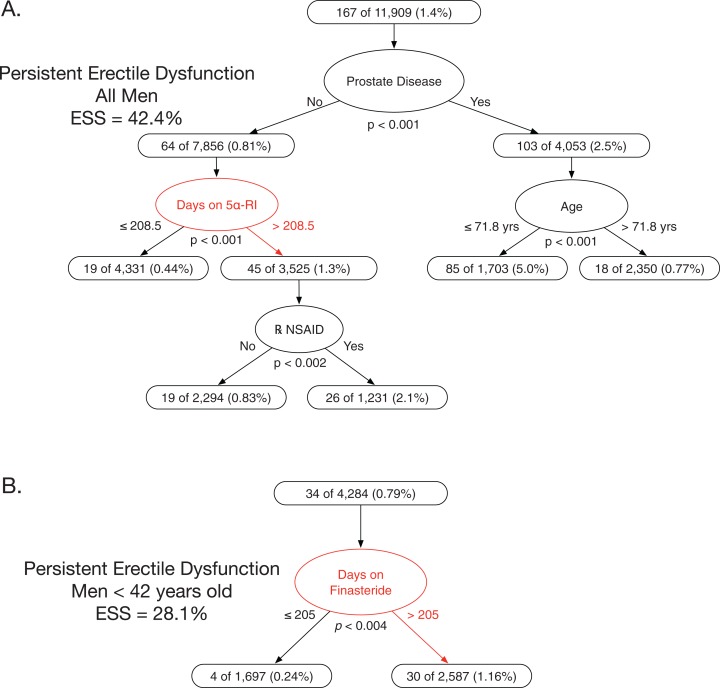
Classification tree analyses for persistent erectile dysfunction in men prescribed 5α-reductase inhibitors. The two classification trees shown are those that predicted new persistent erectile dysfunction in, respectively, all 5α-RI exposed men or in all 5α-RI exposed men <42 years old with greater accuracy than any other possible alternative classification tree given the pool of exposure variables in Table 3. (A) This optimally predictive multivariable model for new persistent erectile dysfunction in all men had four variables: prostate disease, number of days on 5α-RIs, age, and use of prescribed NSAIDs. Men with no prostate disease, >208.5 days on 5α-RIs, and use of prescribed NSAIDs had a 2.1% rate of new persistent erectile dysfunction. The median duration of new persistent erectile dysfunction was 1,348 days. (B) This optimally predictive multivariable model for new persistent erectile dysfunction in finasteride exposed men <42 years old had one variable: number of days on finasteride. Men with >205 days on finasteride had a 1.16% rate of new persistent erectile dysfunction. The median duration of new persistent erectile dysfunction was 1,534 days.

The best multivariable model predicting new PED had four variables: prostate disease, 5α-RI exposure duration, age, and NSAID use (ESS 42.4%, all *p* ≤ 0.002) (Fig. 2A). Among men with no prostate disease, those with longer 5α-RI exposure plus concomitant NSAID exposure had a risk of new PED that was 4.8-fold higher than men with shorter exposure (cutpoint >208.5 days of 5α-RI exposure, NNH = 59.8, all *p* < 0.002).

### New persistent erectile dysfunction in men 16–42 years old and exposed only to finasteride ≤1.25 mg/day

Of 4,284 young men exposed to finasteride ≤1.25 mg/day, and without prior sexual dysfunction and who were evaluated for new PED, 34 (0.79%) developed PED (median 1,534 days after stopping 5α-RI, IQR 651–2,351 days). Of 103 young men with new ED, 34 (33%) had new PED.

#### Univariable predictors for new persistent erectile dysfunction in young men exposed to finasteride ≤1.25

Of the nine significant predictors of new PED in young men, duration of finasteride exposure was the most accurate predictor (cutpoint >205 days of finasteride exposure, NNH 108.2, ESS 28.1%, *p* < 0.004) (Table 3C).

#### Multivariable model for new persistent erectile dysfunction in young men exposed to finasteride ≤1.25

The best multivariable model predicting new PED had one variable: duration of finasteride exposure. Compared to young men with shorter exposure, those young men with longer 5α-RI exposure had a 4.9-fold higher risk (cutpoint >205 days of finasteride exposure, NNH 108.2, ESS 28.1%, *p* < 0.004) (Fig. 2B).

## Discussion

Among an estimated 14 million US men with symptomatic prostatic hyperplasia (National Institute of Diabetes and Digestive and Kidney Diseases, 2015), 5α-RI exposure rose from 4.3% in 1993 to 15.2%, or 2.1 million men, in 2010 (Filson, Wei & Hollingsworth, 2013). An estimated half-million additional men were prescribed finasteride for androgenic alopecia in 2011 (Merck, 2011). Prescribers and patients might reasonably expect that accurate information would be available about the frequency, severity, and persistence of a common adverse effect of a drug approved for marketing more than two decades ago and prescribed to an estimated 2.6 million men annually. However, a meta-analysis of 34 reports of clinical trials of finasteride for androgenic alopecia found inadequate safety reporting and systematic underreporting of adverse events (Belknap et al., 2015), exemplifying a known flaw in the detection or reporting of adverse drug effects in the medical literature (Kostoff, 2016). While a few reports of trials of 5α-RIs for prostatic hyperplasia and lower urinary tract symptoms provide assessments of sexual dysfunction using universal evaluation and validated instruments (Fwu et al., 2014); most rely on spontaneous voluntary reporting for adverse event detection and on global introspection for causality assessment (Belknap et al., 2013); these methods are considered unreliable for detecting and evaluating adverse events in general (Arimone et al., 2007; Koch-Weser, Sellers & Zacest, 1977; Kramer et al., 1985), and sexual dysfunction in particular (Althof et al., 2013; Moore, 2015). Since the introduction of the IIEF in 1997 (Rosen et al., 1997, 1999), the IIEF has been routinely used in clinical trials of PDE_5_Is to assess their efficacy for treatment of sexual dysfunction (Vardi & Nini, 2007). In contrast, the IIEF has rarely been used for assessing sexual adverse effects in clinical trials of 5α-RIs, although many such trials occurred after 1997. An uncontrolled study in 55 men reported that moderate to severe ED (IIEF score <17) was present in 38% of men after 1 month of dutasteride exposure and in 22% of men after 12 months of dutasteride exposure (Chi & Kim, 2011). Despite assertions to the contrary (Singh & Avram, 2014), there is scant available clinical trial data bearing on persistence of sexual dysfunction after 5α-RI exposure. In an oft-cited report on the Prostate Cancer Prevention Trial, some sexual dysfunction variables apparently were not analyzed at all, and adverse effect outcomes were not reported for those subjects who temporarily or permanently discontinued study drug, even though sexual dysfunction was the most common reason for early termination of subjects (Moinpour et al., 2007). In our previous study, we found that 20% of serious adverse events that occur during cancer clinical trials are not reported to an Institutional Review Board and likely were not detected at all by the investigators (Belknap et al., 2013). Such flaws in the design and analysis of clinical trials of 5α-RIs have created a knowledge gap regarding risk of 5α-RI-associated severe sexual dysfunction. Similar flaws in clinical trials resulted in multi-decade delay in recognition of the high frequency of sexual dysfunction associated with thiazide diuretics (Langford et al., 1990), β-adrenergic antagonists (Doumas et al., 2006), and antidepressants (Khazaie et al., 2015).

Our data show that, in a cohort of men exposed to 5α-RIs, the duration of 5α-RI exposure was a more accurate predictor of PED than many known risk factors, including age, hypertension, diabetes mellitus, cigarette smoking, ethanol abuse, obesity, and depression. In our data, confounding by age or extent of healthcare utilization did not account for the increased risk of PED associated with longer 5α-RI exposure duration (Table 3, Figs. 2A and 2B). Also, duration of finasteride exposure proved to be a more accurate predictor of sexual dysfunction than higher dose vs lower dose of finasteride, likely reflecting that finasteride exerts near-maximal inhibition of 5α-DHT synthesis at a dose of 1 mg (Drake et al., 1999; Roberts et al., 1999; Shukla, 2011).

A limitation of our study is the potential for confounding by factors associated with 5α-RI exposure. To address this limitation, our experimental design and statistical analyses included provisions to avoid or mitigate such confounding. We included an extensive set of potential confounders in the analyses, including measures of extent of healthcare utilization, age, BMI, comorbid conditions, and concomitant drugs. We made no a priori assumption about the structure of the predictive multivariable models. To reduce heterogeneity, we used a single-group experimental design (Corrao et al., 2014), excluding men who were not prescribed a 5α-RI. We required both diagnosis of ED and prescription of a PDE_5_I for designation of ED as well as physician description in the narrative for designation of PED. While unmeasured clinical or behavioral attributes may exist, our finding of a consistent effect provides evidence of an intrinsic relationship between duration of 5α-RI exposure and PED. We did not evaluate the extent to which NSAID-associated sexual dysfunction represents either an adverse drug effect (Gleason et al., 2011; Shiri et al., 2006) or confounding by indication for NSAIDs (Patel et al., 2015). We did not evaluate other reported adverse effects associated with 5α-RI exposure, including other sexual effects (infertility, anorgasmia, and sexual anhedonia), genital disorders (Peyronie’s Disease, penile or scrotal numbness, penile or scrotal shrinkage, and infertility), physical effects (gynecomastia, muscle atrophy, thinning, and drying of skin), cognitive disorders (memory impairment, slowed cognition, and confusion), or psychological disorders (anxiety, depression, anhedonia, and insomnia) (Ganzer, Jacobs & Iqbal, 2015).

The lower rate of detection of sexual dysfunction in older men likely reflects diminished disclosure by older men and lower likelihood of inquiry about sexual health by their physicians (Loeb et al., 2011). The predictor cutpoints for 5α-RI exposure duration do not establish a safe threshold for exposure duration. Our use of a single-group design for the primary analysis means that the observed NNH must be considered as an upper bound, as all men in the study cohort were exposed to 5α-RIs. As our data source was derived from an existing EMR system, detection of sexual dysfunction was necessarily dependent on what clinicians entered into the medical record. Evaluations using standardized instruments, such as the IIEF (introduced in 1997), were not routinely recorded in the source medical record and thus was not reliably available for our study. We expect that a clinical trial using randomization, placebo-control, universal evaluation, and a validated measure of ED would give a higher attributable risk and therefore a lower NNH.

Androgenic alopecia and prostatic hyperplasia are chronic, non-life-threatening conditions. In 1994, the International Conference on Harmonization (ICH) provided a guideline for assessment of the safety of drugs being developed to treat chronic, non-life-threatening conditions. This guideline recommends that a cohort of 300–600 subjects be exposed to the new drug for six months, and that 100 subjects be exposed for 1 year. This guideline does not directly address the evaluation of resolution or persistence of an adverse drug event (ICH, 1994). Although severe sexual dysfunction was a foreseeable consequence of 5α-reductase inhibition, it is not clear if the pivotal clinical trials for finasteride and dutasteride included assessment for persistent sexual dysfunction or other severe sexual adverse effects. If there was meaningful assessment of persistent sexual dysfunction in humans during the clinical development of the 5α-RIs, this does not appear to have been reported in the medical literature, the FPI, or other publicly accessible sources.

In conclusion, among men with 5α-RI exposure, duration of 5α-RI exposure was a more accurate predictor of PED than all other assessed risk factors except prostate disease and prostate surgery. Among young men with 5α-RI exposure, duration of 5α-RI exposure was a more accurate predictor of PED than all other assessed risk factors. For each 108 young men exposed for >205 days to the finasteride dose typically used for androgenic alopecia (≤1.25 mg/day), one additional young man experienced PED when compared to those men with shorter exposure. The median duration of PED in young men was 1,534 days. We expect that our finding of an association between debilitating sexual dysfunction and exposure to finasteride or dutasteride will be of particular interest to prescribers and patients considering medical management of androgenic alopecia or symptomatic treatment of prostatic hyperplasia.

## Additional Information and Declarations

### Data Deposition

The following information was supplied regarding data availability: The raw data was provided for review, but cannot be made publicly available because, though it has been de-identified, it is possible to re-identify the patients and would compromise their privacy.
